# High interleukin-4 expression and interleukin-4 gene polymorphisms are associated with susceptibility to human paracoccidioidomycosis

**DOI:** 10.1590/0074-02760150197

**Published:** 2015-09

**Authors:** Mônica Sawan Mendonça, Terezinha S Peraçolli, Mário León Silva-Vergara, Sílvio C Ribeiro, Rafael Faria Oliveira, Rinaldo Poncio Mendes, Virmondes Rodrigues

**Affiliations:** 1 Universidade Federal do Triângulo Mineiro, Laboratório de Imunologia, Uberaba, MG, Brasil; 2 Universidade Estadual Paulista, Faculdade de Medicina de Botucatu, Botucatu, SP, Brasil

**Keywords:** gene polymorphism, IL-4, Paracoccidioides brasiliensis, paracoccidioidomycosis

## Abstract

Paracoccidioidomycosis (PCM) is caused by dimorphic fungi from the*Paracoccidioides brasiliensis* complex. Previous studies have demonstrated that the severity of disease is associated with a T-helper 2 immune response characterised by high interleukin (IL)-4 production. In the present study we analysed two polymorphisms in the* IL-4 *gene (-590 C/T and intron-3 microsatellite) in 76 patients with PCM and 73 control subjects from an endemic area. The production of IL-4 by peripheral blood mononuclear cells after antigen or phytohaemagglutinin stimulation was determined by ELISA. A significant correlation was observed between the RP2/RP2 intron-3 genotype and infection with *Paracoccidioides sp. *(p = 0.011), whereas the RP1/RP1 genotype was correlated with resistance. No significant correlation was observed for the* IL-4 *promoter polymorphism. Furthermore, the low IL-4 expression observed in the control group compared with patients was associated with the RP1/RP1 genotype. These results suggest that *IL-4*polymorphisms might be associated with the ability of the host to control* Paracoccidioides sp. *infection. The relevance of this polymorphism is supported by the observation that patients with disease produce high levels of IL-4 following mitogen or antigen stimulation. The* IL-4 *gene is located in the cytokine cluster region of chromosome 5 where other polymorphisms have also been described.

Paracoccidioidomycosis (PCM) is the most prevalent systemic mycosis in Latin America and is endemic in Brazil, Argentina, Venezuela and Colombia (Restrepo 1985, Blotta et al. 1999). The disease is caused by the dimorphic fungi *Paracoccidioides brasiliensis*and *Paracoccidioides lutzii. *After invasion, the fungi are either immediately destroyed or overcome the local defences of the host and begin multiplication, thereby causing initial injury. In humans, the disease is characterised by a broad spectrum of clinical manifestations that range from localised mucocutaneous lesions to widespread manifestations involving the mononuclear phagocyte system (Shikanai-Yasuda et al. 2006). Patients with active disease present high levels of specific antibodies, but no delayed-type hypersensitivity reaction to specific antigen stimulation. These properties are closely related to the outcome of infection and are used for the clinical follow-up of treated patients and as criteria for cure of the disease (Marques 2012).

Cytokines are pleiotropic molecules that regulate many aspects of the immune response and inflammatory reactions (Fitzgerald et al. 2001). T-helper (Th) cells are the major source of regulatory cytokines and can be divided into at least two distinct polar subpopulations according to their cytokine pattern (Bernard et al. 1997). In humans, Th1 cells produce interferon (IFN)-γ, tumour necrosis factor (TNF)-β and TNF-α, whereas Th2 cells produce interleukin (IL)-4, IL-5 and IL-13. Because of their strong influence on immune effector mechanisms, the preferential activation of one population or another is associated with resistance/susceptibility to infectious diseases.

Previous studies have demonstrated that PCM is associated with a Th2 immune response that is characterised by high IL-4 production after antigen or phytohaemagglutinin (PHA) stimulation (Mello et al. 2002, Cavassani et al. 2011). Because certain gene polymorphisms are able to modulate their expression, genetic variants may be associated with resistance or susceptibility to infectious diseases (Bozzi et al. 2006). *IL-4* is located on chromosome 5 in the 5q31-q33 region, close to the genes of many other regulatory cytokines (Fitzgerald et al. 2001). *IL-4* gene polymorphisms have been described in association with some noninfectious diseases, such as rheumatoid arthritis, asthma and atopy (Burchard et al. 1999, Cantagrel et al. 1999, Rosenwasser 1999, Nie et al. 2013), and with infectious diseases, such as dental infection (Michel et al. 2001, Kang et al. 2003, Pontes et al. 2003, Scarel-Caminaga et al. 2003), cases of malaria (Verra et al. 2004) and human immunodeficiency virus (HIV)-1 infection (Nakayama et al. 2002, Modi et al. 2003).

In this study, we selected IL-4 as a candidate gene due to its biological properties in the regulation of immune responses, some of which have been observed in PCM patients, including high serum levels of immunoglobulins and the absence of delayed type hypersensitivity to *P. brasiliensis* antigens. Furthermore, we and others have observed that IL-4 was produced at high levels by peripheral blood mononuclear cells (PBMC) after antigen or PHA stimulation (Mello et al. 2002, Cavassani et al. 2011). We analysed two polymorphisms, one in the promoter region and the second in intron-3 of the* IL-4* gene, both in patients with PCM and in control subjects from an endemic area as well as the expression of IL-4 in PBMC supernatants after stimulation with *P. brasiliensis* crude antigens and PHA.

## SUBJECTS, MATERIALS AND METHODS

*Subjects* - The study was conducted at two Brazilian centres: the General Hospital of Federal University of Triângulo Mineiro (UFTM) and the University Hospital of Botucatu Medical School, São Paulo State University (UNESP). To be eligible for the study, patients had to give informed consent and to have confirmed PCM. The study protocol was reviewed and approved by the Research Ethical Committee of UFTM. All participants provided written informed consent to participate in this study.

Seventy-six patients (20 women and 56 men), aged 33.2 ± 14.1 years, with a diagnosis of PCM were included in this study. The diagnosis was confirmed *via*the identification of typical *P. brasiliensis *yeast forms through mycological examination, cell-block preparation and/or histopathological examination of clinical specimens. Patients were included regardless of the phase of disease, i.e., active (before treatment), under treatment and cured with or without sequelae. The control group consisted of 73 healthy subjects from endemic areas (20 women and 53 men), aged 33.9 ± 12.0 years, who had a positive skin test to paracoccidioidin and a negative reaction to histoplasmin and who showed no present or past symptoms that were compatible with PCM.

The skin tests were performed using paracoccidioidin and histoplasmin. Paracoccidioidin was prepared as sterile polysaccharide antigen at the Department of Pathology, Botucatu Medical School, UNESP, from a pool of several*Paracoccidioides *sp. strains in the yeast form. Histoplasmin was prepared as a sterile culture filtrate from the mycelial growth of*Histoplasma capsulatum* var. *capsulatum* strains B-679 and A-811 at the Evandro Chagas Clinical Research Institute, Oswaldo Cruz Foundation. The tests were carried out according to classical protocols (Gascan et al. 1991, Rosenwasser 1999).

*IL-4 expression* - Blood samples (10 mL) were collected from the patients and PBMCs were isolated by Ficoll-Paque centrifugation (400*g*, 20 min at room temperature), washed three times in RPMI medium and resuspended in RPMI (Gibco, USA) supplemented with 50 mM 2-ME, 2 mM L-glutamine, 40 µg/mL gentamicin and 10% foetal calf serum (complete medium).

For supernatant production, 2 × 10^6^ cells per well per mL were cultured in a 24-well microplate in the presence of medium alone, 5 µg/mL PHA, or 5 µg/mL cell-free antigens from a pool of several *Paracoccidioides *spp strains in the yeast form. The plates were incubated at 37ºC in a 5% CO_2_atmosphere for 48 h and the supernatants were collected, centrifuged and stored at -70ºC until IL-4 production analysis. IL-4 was titrated by ELISA in 96-well microplates (Nunc, Denmark) using commercial monoclonal antibody pairs (Mabtech, Sweden). The sensitivity of the assay was 2 pg/mL.

*IL-4 gene polymorphism* - Blood samples (10 mL) were collected from the patients and leukocytes were purified by red blood cell lysis. Genomic DNA was purified using the DNAzol reagent. The two regions of interest were amplified by polymerase chain reaction (PCR) using the following primers: 5′-AGGTGAAAGGGGAAAGC-3′ and 5′-CTGTTCACCTCAACTGCTCC-3′ (Bozzi et al. 2006) for analysis of the intron-3 microsatellite and 5′-ACTAGGCCTCACCTGATACG-3′ and 5′-GTTGTAATGCAGTCCTCCTG-3′ (Fitzgerald et al. 2001) for the SNP at -590. In the case of the C/T substitution at position -590, the PCR product was digested with *Ava*II. The resulting products were analysed by polyacrylamide gel electrophoresis.

*Statistical analysis* - Genotype frequencies were analysed statistically by the chi-square test, with the level of significance set at 5% (p < 0.05). The association of the IL-4 levels with disease or IL-4 genotypes was analysed using the Mann-Whitney *U* test. Statistical analysis was performed using the GraphPad Prism software (GraphPad Software Inc, USA).

## RESULTS

*Intron-3 polymorphism* - The intron-3 polymorphism of the*IL-4* gene was determined in 149 subjects (76 patients and 73 controls) and three genotypes (RP2/RP2, RP2/RP1 and RP1/RP1) were detected. A significantly lower frequency of the RP1/RP1 genotype was observed in patients (5.2%) compared with the control group (23.3%). In contrast, the frequency of the RP2/RP2 genotype was 53.9% in patients and 37% in controls [p = 0.0042; odds ratios (OR): RP2RP2 x RP1RP1 = 6.454; 95% confidence interval (CI): 1.958-21.28]. The frequency of the heterozygous RP1/RP2 genotype was similar in both patients and controls (Table I).

**TABLE I:** Distribution of the frequency of genotypes RP1/RP1, RP2/RP1 and RP2/RP2 in controls individuals and patients infected with*Paracoccidioides *sp.

Genotypes	RP1/RP1 n (%)	RP2/RP1 n (%)	RP2/RP2 n (%)	Total n
Controls	17 (23.3)	29 (39.7)	27 (37)	73
Patients	4 (5.2)	31 (40.7)	41 (53.9)	76
Total	21 (14.09)	60 (40.27)	68 (45.64)	149

p = 0.0042; odds ratios: RP2RP2 x RP1RP1 = 6,454; 95% confidence interval: 1.958-21.28.

The association of RP2 with disease was confirmed by the relative risk of 6.454 (CI: 1.958-21.28) when comparing RP2RP2 with RP1RP1. The frequencies of the RP1 and RP2 alleles were 38.23 and 57.65%, respectively, in patients and 61.77% and 42.35% in the control group, thus demonstrating an association of the RP2 allele with disease (χ^2^ = 10.12; p = 0.0015), with a relative risk of 2.01 (CI: 1.348-3.589).

*-590 C/T promoter polymorphism* - The -590 C/T polymorphism in the promoter region of the *IL-4* gene was analysed in 149 subjects (76 patients and 73 controls) and three genotypes (CC, TC and TT) were detected. No significant correlation was found between these three genotypes and infection with*P. brasiliensis*. The frequency of genotype CC was 20.54% in controls and 10.52% in patients. Genotype TT was present in 50.68% of the controls and 60.53% of the patients. The frequency of the heterozygous genotype (CT) was similar in the two groups (Table II). No significant correlation was observed in the genotypes or in the allele frequency between patients and controls.

**TABLE II:** Distribution of the frequency of genotypes CC, CT and TT in controls individuals and patients infected with *Paracoccidioides*sp.

Genotypes	CC n (%)	CT n (%)	TT n (%)	Total n
Controls	15 (20.54)	21 (28.76)	37 (50.68)	73
Patients	8 (10.52)	22 (28.95)	46 (60.53)	76
Total	23 (15.44)	43 (28.86)	83 (55.70)	149

p = 0.2154.

*IL-4 expression* - Significantly higher levels of IL-4 were observed in supernatants from patients with disease compared with controls (p < 0.01) (A in Figure). After PHA stimulation, the IL-4 levels ranged from 32-256 pg/mL (median: 74 pg/mL) in the supernatants of patients, whereas these levels ranged from undetectable to 41 pg/mL (median: 10 pg/mL) in the control group. In supernatants stimulated with cell-free antigen, the IL-4 levels ranged from 26-66 pg/mL (median: 40 pg/mL) in patients and were below the detection limit in the control group. The analysis of IL-4 production by PBMC after PHA stimulation and grouped according to intron-3 genotype demonstrated that the RP1-1 genotype produced significantly less IL-4 than did RP2-1 and RP2-2 (p < 0.05) (B in Figure).

## DISCUSSION

IL-4 has multiple immune response-modulating functions, including the induction of IgE production by B lymphocytes and the differentiation of precursor Th cells toward the Th2 subset that mediates humoral immunity. IL-4 also acts as the main antagonist of IFN-γ and thus inhibits the activation of macrophages and cell-mediated reactions (Nakayama et al. 2002). A study analysing the cellular immune response in patients with PCM reported a strong Th2 response after stimulation with PHA, which was characterised by high levels of IL-4 and IL-5 (Mello et al. 2002). The resistance to infection is mainly determined by cellular immunity, more specifically by the effective cooperation between T lymphocytes and macrophages (Franco et al. 1989, Rigobello et al. 2013). In contrast, a Th2 response characterised by high levels of IL-4 has been associated with infection and the development of severe forms of disease (Mello et al. 2002). Elevated levels of IL-4 associated with very low levels of IFN-γ seem to lead to the development of an unfavourable specific immune response, such as that observed in patients with the multifocal form of PCM. The results obtained for patients with the unifocal form support this idea, based on the observation of reduced levels of IL-4, elevated levels of IFN-γ and a less severe course of the disease (Mello et al. 2002). It is still unclear whether IL-4 and IFN-γ are responsible for susceptibility or resistance, respectively, to infection with*P. brasiliensis*. However, such susceptibility and resistance to infectious diseases are likely the result of interactions among multiple factors rather than a single factor (Abel & Dessein 1997).

**Figure:**
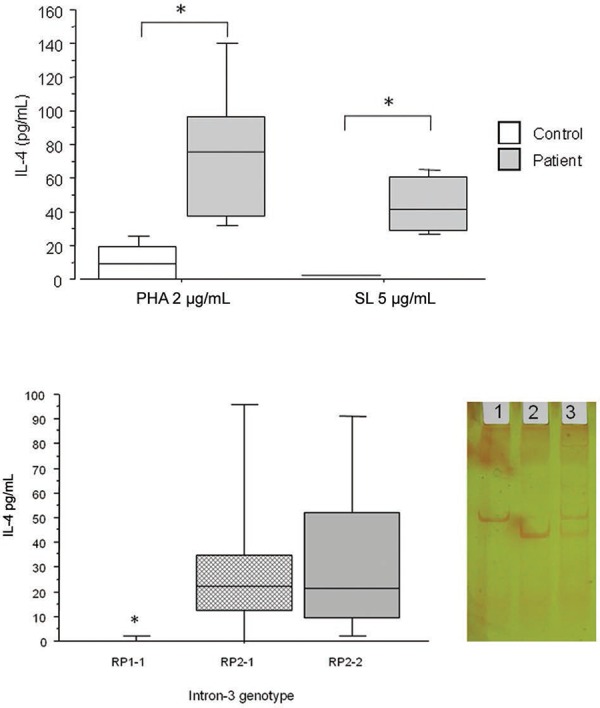
Production of interleukin (IL)-4 by peripheral blood mononuclear cells from patients and control subjects stimulated with phytohaemagglutinin (PHA) or crude soluble *Paracoccidioides brasiliensis* antigens (A) or on all casuistic (patients and controls) grouped according to intron-3 genotype (B). Horizontal lines represent the median, bars represent the 25-75% percentiles and vertical line the 10-90% percentiles. Asterisk mean significance with p < 0.05 Mann-Whitney *U* and Kruskal-Wallis. Lateral panel of B: a representative polyacrylamide gel electrophoresis of intron-3 polymorphism. Line 1: PR2-2; 2: RP1-1; 3: RP2-1. RP1 correspond to 183 bp and RP2 correspond to 253 bp DNA fragments. SL: streptolysin.

In this study, we investigated two *IL-4* gene polymorphisms in patients with PCM and control subjects from an endemic area: the dimorphic intron-3 polymorphism, which is represented by a microsatellite in which RP1 corresponds to two repetitions in the 70-bp sequence and RP2 to three repetitions in the same sequence and a polymorphism in the promoter region, which corresponds to a C/T substitution at position -590 (Cantagrel et al. 1999). In the sample studied, the frequency of the RP1/RP1 genotype was four times higher in the control group than in the patient group (23.3% vs. 5.2%). In contrast, the RP2/RP2 genotype was observed in 37% of the controls and 53.9% of the patients. The frequency of the heterozygous genotype (RP1/RP2) was similar in the two groups. These data indicate a correlation between the presence of the RP2/RP2 genotype and infection, whereas the RP1/RP1 genotype is correlated with resistance. With respect to the promoter polymorphism, we did not observe a significant difference between patients and controls.

Furthermore, the present study provides evidence that the expression of IL-4 is up-regulated in patients after antigen and mitogen stimulation, in agreement with data previously published by our group (Mello et al. 2002) and with the findings reported by Cavassani et al. (2011). These data are also supported by the observation that PCM patients exhibit high levels of IgE-specific antibodies (de Britto & Franco 1994). Moreover, this study provides evidence that PBMC from subjects bearing the RP1-1 genotype at intron-3 are associated with disease protection (OR: RP2RP2 x RP1RP1 = 6.454) and produce less IL-4 than do the RP2-1 or RP2-2 genotypes.

However, the small sample size of this study is a limitation. We thus consider that PCM is a lowly endemic disease; all control subjects were from the same endemic area and, upon selection, presented signs of *Paracoccidioides *sp. exposure based on a positive skin test to paracoccidioidin or the reactivity of serum antibodies to *Paracoccidioides *sp. antigens.

In humans, the gene encoding IL-4 is located on chromosome 5 in the 5q31-q33 region, which is the so-called cytokine cluster, where other genes important for the immune system, such as IL-5, IL-9, IL-12, IL-13 and granulocyte-macrophage-colony-stimulating factor, are also found. These cytokines play an important role in the regulation of Th1/Th2 immune responses (Fitzgerald et al. 2001). Case-control studies have provided evidence for a correlation of the intron-3 *IL-4*promoter polymorphisms with various diseases, such as rheumatoid arthritis (Cantagrel et al. 1999), respiratory and infectious disorders in children (Zhu et al. 2000, Segal & Hill 2003, Steinke et al. 2003) and early-stage dental infections or susceptibility to dental plaques caused by microorganisms (Michel et al. 2001). The protective effect of the *IL-4* promoter polymorphism in patients with HIV-1 was demonstrated by the observation that this polymorphism was correlated with viral load in patients in the progressive phase of the disease (Nakayama et al. 2002). This same polymorphism was associated with viral persistence in hepatitis B virus infection (Saxena et al. 2014). In renal cell carcinoma, this promoter polymorphism was associated with disease development and survival (Kleinrath et al. 2007). With respect to diseases caused by fungi, a correlation between the *IL-4* gene polymorphism and high levels of vaginal IL-4 was demonstrated in recurrent vulvovaginal candidiasis (Babula et al. 2004). Furthermore, an association was reported between chronic disseminated candidiasis in adult acute leukaemia and the *IL-4* promoter polymorphism (Choi et al. 2003).

To our knowledge, no functional studies have been performed on the*IL-4* gene polymorphisms analysed here using construction genes. However, the present results indicate a significant correlation between the intron-3 polymorphism and the occurrence of disease. The marked production of this cytokine was observed in patients with PCM, in agreement with previous findings reported by our group (Mello et al. 2002) and others (Cavassani et al. 2011). In addition, low IL-4 levels were observed in subjects with the polymorphic genotype RP1-1 at intron-3. The *IL-4* gene is located in the cytokine cluster on chromosome 5, where other genes such as *IL-5*,*IL-9*,* IL-12* and *IL-13* are also found and all of these genes have a potential regulatory role in the immune response.*IL-4*, *IL-13* and *IL-5* genes were found to be regulated coordinately by several long-range regulatory elements in a greater than 120 kb range on chromosome 5. Thus, the correlation observed might be attributed to another gene located in the region whose polymorphism is in linkage disequilibrium with the polymorphism studied here. Another study evaluating IL-10 and TNF-α gene polymorphisms in PCM demonstrated an association between the IL-10 polymorphism and disease (Bozzi et al. 2006). In the present study, we analysed a larger number of patients and the control subjects were all from endemic areas and were considered to be exposed to*Paracoccidioides *sp. because they presented a positive reaction to fungal antigens.

The present results indicate a significant correlation between the presence of the RP2/RP2 genotype and PCM, whereas the RP1/RP1 genotype is correlated with resistance and with low production of IL-4. These data support the importance of IL-4 for the determination of susceptibility to *Paracoccidioides *sp. and open new perspectives for the development or introduction of new therapies for PCM.
